# Risk of Suicide and Dysfunctional Patterns of Personality among Bereaved Substance Users

**DOI:** 10.3390/ijerph14030316

**Published:** 2017-03-20

**Authors:** Laura Masferrer, Beatriz Caparrós

**Affiliations:** 1 Public Drug Centre, Cas Teresa Ferrer-CAS Ripoll, Institut d’Assistència Sanitària (IAS), 17003 Girona, Spain; 2 Department of Psychology, University of Girona, 17071 Girona, Spain; beatriz.caparros@udg.edu

**Keywords:** risk of suicide, personality disorders, substance use disorder

## Abstract

*Background*: Research has shown that suicide is a phenomenon highly present among the drug dependent population. Different studies have demonstrated an upraised level of comorbidity between personality disorders (PD) and substance use disorders (SUD). This study aimed to describe which PDs are more frequent among those patients with a risk of suicide. *Methods*: The study was based on a consecutive non-probabilistic convenience sample of 196 bereaved patients attended to in a Public Addiction Center in Girona (Spain). Sociodemographic data, as well as suicide and drug related characteristics were recorded. The risk of suicide was assessed with the Spanish version of “Risk of suicide”. Personality disorders were measured with the Spanish version of Millon Multiaxial Clinical Inventory. *Results*: The PDs more associated with the presence of risk of suicide were depressive, avoidant, schizotypal and borderline disorders. However, the histrionic, narcissistic and compulsive PDs are inversely associated with risk of suicide even though the narcissistic scale had no statistical correlation. *Conclusions*: The risk of suicide is a significant factor to take into account related to patients with SUD and especially with the presence of specific PDs. These findings underline the importance of diagnosing and treating rigorously patients with SUD.

## 1. Introduction

Approximately 800,000 people die due to suicide around the world every year [1,2]. Suicidal risk is a very complex behavior that is influenced by interacting biological, genetic, psychological, social, environmental and situational factors, as several authors have described [3,4].

The link between the risk of suicide and substance use disorders (SUD) is well documented [5,6,7,8]. Li and collaborators [9] showed that the risk of suicide was 7.5 times higher in males and 11.7 times higher in females with a mental or SUD compared to males and females with no disorder. In another recent study among the SUD population, Masferrer et al. [10] found that 61.2% of 196 bereaved SUD patients reported a risk of suicide in a large study focused on describing related variables of risk of suicide among bereaved addicted patients. Taking into account the connection between the risk of suicide and SUD, our interest is to analyze two important variables, personality disorder and risk of suicide, because it could have an important influence on the particular and complex association between these constructs. Our interest is to study the risk of suicide as associated with the comorbid dysfunctional patterns of personality in SUD patients. 

Personality Disorders (PDs) are defined as inflexible and maladaptive personality traits that are exhibited in a wide range of personal and interpersonal contexts [11]. The Diagnostic and Statistical Manual of Mental Disorders 5 [11], in section II, defines PDs as categorical entities. However, based on the discussion of early research [12,13,14,15,16], PDs have been characterized as dimensional constructs related to a framework that provides a unified model of psychopathology established on shared personality traits. In fact, Diagnostic and Statistical Manual of Mental Disorders DSM-5, in section III, proposes a dimensional alternative model. In the framework of this dimensional approach of psychopathology, Millon’s integrative model of personality disorders [17,18] proposes an explanation of the structure of personality styles on the background of ecological adaptation. Millon classifies personality disorders in accordance with four main dimensions: Personalities with difficulties in taking pleasure (i.e., with schizoid, avoidant or depressive disorders), personalities with interpersonal problems (with dependent, histrionic, narcissistic or antisocial disorders), personalities with intrapsychic conflicts (with sadistic, compulsive, negativistic or masochistic disorders) and personalities with structural deficits (with schizotypal, borderline or paranoid disorders). The latter three pathological personality patterns (schizotypal, borderline and paranoid) represent, in terms of Millon’s theory, more advanced stages of personality pathology and structural impairment. 

PDs can seriously influence the course, prognosis and the treatment outcomes of SUDs [19]. Reporting a diagnosis of PD is linked with greater impairments as well as a lower quality of life [20,21]. The presence of PD is a greatly prevalent comorbid disorder among substance users [22,23,24,25]. In fact, different studies have demonstrated a high level of comorbidity between PD and SUD [26,27,28,29,30,31]. As Krueger and Eaton [32] stated, comorbidity is the rule not the exception. In this regard, Gonzalez [33] found a prevalence of any personality disorder of 42% among a sample of 53 alcohol and drug dependent inpatients. Colpaert and collaborators [27] reported a rate of 42.6% for at least one PD among 274 patients admitted to a residential substance abuse treatment. Casadio et al. [26] described a rate of 62.2% among addiction outpatients. Moreover, Verheul [19] concluded that rates of PDs among the drug dependent population are four times higher than among the general population. 

Bearing in mind the negative impact of PDs and the potential risk of suicide, this study aimed to describe which dysfunctional patterns of personality are more frequent among those SUD patients with a risk of suicide and which dysfunctional patterns of personality are more frequent among those without a risk of suicide.

## 2. Material and Methods

The current study is part of wider research. The main goal of this research was to describe the complicated grief symptomatology among a sample of 196 bereaved SUD patients. For more information, see Masferrer et al. [10].

### 2.1. Participants

The current research was based on a consecutive non-probabilistic convenience sample of individuals (*n* = 196) attended the Public Addiction Treatment Centre in Girona (Catalonia, Spain). To join the study, patients had to meet the following three inclusion criteria: (a) they had a diagnosis of substance use disorder (SUD) (alcohol, cocaine or heroin dependence) according to the 4th revised edition of Diagnostic and Statistical Manual of Mental Disorders (DSM-IV-TR) criteria; (b) loss of a significant person (family, best friend or partner) at some time in their life, but at least a year previously to the interview; and (c) abstinence during the last month to avoid any toxic effects of drugs. The majority of patients (78.1%) were male, more than a third (37.2%) were married or with a partner. Related to the main drug diagnosis, the majority of the patients (68.9%) reported alcohol dependence, 18.4% heroin dependence and 12.8% cocaine dependence. 

### 2.2. Measures

For the assessment of the risk of suicide, we used the Spanish version of the *Risk of Suicide* (RS) from Plutchick et al. [34]. The RS could discriminate between individuals and patients with no suicide attempts and those having a history of them. It consists of 15 items with dichotomous responses (yes/no). The RS embraces issues about previous attempts, ideation intensity of current feelings of depression and hopelessness, and other aspects of the attempts. The total score is obtained by summing all items (maximum score 15). The cut-off suggested by the authors of the Spanish version [35] was 6. Internal consistency of the test is 0.90. 

Personality disorders were measured by the *Millon Multiaxial Clinical Inventory* [36], the Spanish translation by Cardenal and Sánchez-López [37]. The MCMI-III consists of 175 items with dichotomous answers (true/false), a self-report questionnaire that measures 11 clinical personality patterns, 3 traits of severe personality pathology, 7 syndromes of moderate severity, 3 severe syndromes and a validity scale and 3 modifying indices. The PDs scales cover major diagnostic criteria of DSM-IV-TR. We adopted the most conservative criteria with scores equal or greater than 85 to determine the presence of the PDs. Clinical personality patterns (schizoid, avoidant, depressive, dependent, histrionic, narcissistic, antisocial, sadistic, compulsive, negativistic and masochistic) and 3 traits of severe personality pathology (schizotypal, borderline, and paranoid) were used in the current research.

### 2.3. Procedure

Those patients who met the three inclusion criteria were informed by their therapist about potential participation in the study. The research procedure consisted of a single visit with a psychologist who administered the questionnaires included in the study protocol. All patients were previously informed about the study procedure as well as terms of confidentiality. Informed consent was obtained from all participants and the protocol was approved by the Institutional Ethics and Research Review Board of the Institut Assistència Sanitària (IAS) (No. S041-779). 

### 2.4. Statistical Analyses

The risk of suicide was measured as the relative frequency and the 95% level of confidence of participants above the RS cut-off point. In order to compare individuals with and without risk of suicide, we performed a bivariate analysis of the PD scores of the patients according to the risk of suicide. According to the nonparametric Kolmogorov Sminov test, the different Millon’s scales did not follow a normal distribution and the significance was below 0.05 (except for the negativist scale which is the only scale with normal distribution with a signification of 0.072). Due to the small group of PDs, we used a non-parametric statistical test. When PDs were defined as categorical variables, we used a Fischer Exact test and when PDs were defined as a dimensional variable, we used *U* Mann Whitney and Spearman’s correlation. A multiple regression analysis was performed to determine which dysfunctional patterns of personality were associated with the risk of suicide. The results are expressed as absolute numbers, percentages, as well as the mean and standard deviations. A statistical significance of 0.05 was used to compare hypotheses. Data processing and analysis were performed using the SPSS statistical program version 21.0 for Windows (IBM Corp., Armonk, NY, USA).

## 3. Results

### 3.1. Dysfunctional Patterns of Personality 

Taking into account that we adopted the most conservative criteria of Millon’s scoring for describing the presence of PD (scores equal or greater than 85), the presence of any PD among the sample was 29.4%. Describing the occurrence according to each PD, those PD with higher frequency were compulsive (7.1%) and narcissistic (7.1%), followed by antisocial (4.6%) and sadistic (3.1%). Twenty-four percent of the patients reported a presence of one PD and only 1.5% two PD. On the other hand, avoidant, dependent and masochistic were not present as a disorder. 

The first objective of the study was determine which PDs (scoring equal or greater than 85) are more frequent among those patients with a risk of suicide and which PDs are more frequent among those without a risk of suicide. Bearing in mind that the number of PDs was small, we performed a non-parametric statistical test. The results of relationship between PDs as categorical variables and the risk of suicide are set out in Table 1, in which the Fischer Exact test was carried out. What stands out in the first table is that, in the risk of suicide group, there is a higher presence of schizoid, depressive, narcissistic, antisocial, sadistic, schizotypal, borderline and paranoid cases, although the differences between the groups are not statistically significant in any case. The histrionic and compulsive disorders are more present, in a significant way, in the no risk of suicide group. If we compare the direct scores of the different scales of PDs, significant differences are shown in the scores of all scales except in narcissistic through the *U* Mann Whitney analysis (Table 2). Furthermore, those patients grouped in the risk of suicide presented a higher mean than those without risk, not including histrionic, narcissistic, compulsive and borderline.

### 3.2. Analysis of the Relationship between Symptomatology of PDs and Risk of Suicide

In addition, the relationship between the risk of suicide and symptomatology of PDs was investigated using Spearman’s correlation coefficient (Table 3). The different scores of PDs showed a significant association with the risk of suicide, with the exception of the narcissistic. The three higher and stronger correlations were borderline (*r* = 0.703), depressive (*r* = 0.628) and masochistic (*r* = 0.529). Otherwise, results indicated an inverse correlation between the risk of suicide and histrionic, narcissistic and compulsive scales, even though the narcissistic scale did not have any statistical correlation.

### 3.3. Predictive Characteristics of Risk of Suicide

Another main objective of the study was to determine which PDs were associated with the risk of suicide. Thus, in order to describe this, a multiple regression, in which scores of the risk of suicide, as dimensional variables, were the dependent variable, was performed while sociodemographic, suicide-related characteristics (age, gender, education, marital status and patient’s suicide attempt) and dysfunctional patterns of personality were considered independent variables. The results were presented in Table 4. Those dysfunctional patterns of personality defined also as dimensional variables associated with the risk of suicide were avoidant, depressive, schizotypal and borderline.

## 4. Discussion

Almost one third of our sample (29.4%) reported some PDs. Therefore, PDs are quite frequent among the current SUD sample, which is in agreement with those results obtained by previous studies [23,24,25]. Comorbid PD and SUD represent a robust determinant of elevated suicide risk [38]. However, the scales in which there are any cases with a score above 85 were avoidant, dependent and masochistic.

The primary goal of this study was to determine which dysfunctional patterns of personality are more frequent among those patients with a risk of suicide and which PDs are more frequent among those without a risk of suicide. There are very few patients who reported high scorings of PD in the sample. When we have dichotomized PD variables in “presence of PD” or “absence of PD”, it can be seen that there are very few cases. The differences between the group of “risk of suicide” and “no risk of suicide” are not significant in the different PD, except for histrionic, sadistic and compulsive. However, it should be noted that more PDs were associated with the risk of suicide than without the risk of suicide. Histrionic and compulsive are more frequent in the no risk of suicide group, and sadistic in the risk of suicide group. For this reason and following the theoretical approach of early research [13,15,16], we wanted to analyze more thoroughly the different PD scales in a dimensional way. Consistent with our expectations, reporting a high scoring in PDs’ scales is linked with the risk of suicide, except in the cases of the histrionic, narcissistic and compulsive scales, although histrionic and compulsive are the only scales in which the differences are statistically significant. Specifically, when analyzing how PDs scoring and the risk of suicide perform, borderline, depressive and masochistic are the three scales with a higher association with risk of suicide. These results confirm the important role of PDs as risk factors for suicide as other studies suggested [39,40].

An important finding was that the narcissistic, compulsive and histrionic scales had an inverse correlation with the risk of suicide. A potential explanation for these findings might be that stating personality traits of these scales could be defined as protective factors against suicidal risk. Nowadays, some personality characteristics from those PDs are greatly accepted and promoted in Western society [41]. These results lead us to reflect on previous investigations carried out with the MCMI [37], in which a curved model of the narcissistic, histrionic and compulsive scales is considered, meaning that it is the low and the high scores that indicate non-adaptation, whereas intermediate levels on these scales would reflect adaptive patterns, unlike what happens in relation to other scales [41]. Turning next to the narcissistic scale, it was the only PD with no present relationship with the risk of suicide. This finding indicates that the narcissistic PD appears to be a distinct group among cluster B personality disorders related to suicidal risk but this outcome is contrary to that of Pompili and his collaborators [3], who found that individuals with cluster B personality disorders have a greater risk of dying by suicide. At this point, it should be taken into account that presenting specific personality traits does not necessarily entail negative consequences in relation to the presence of mental health problems but reporting many personality traits could be associated to a general dysfunctional pattern of personality, so could be linked to risk of suicide. Therefore, it is important to be aware of differences between dimensional analysis (direct scoring) and categorical analysis (presence–absence of PD) in order to describe the specific characteristics that may be more protective for those who make up the PD. As some authors stated [42], each case has a profile that emerges from quantitative variations and different levels ranging from normal to pathology without the need to cut-off that could be artificial.

In order to identify which PDs were linked to the risk of suicide, a multiple regression analysis was conducted. Talking about the sociodemographic variables related to the risk of suicide, marital status was the only relevant characteristic. Being separated or divorced and widowed was statistically significant with the risk of suicide. These results are in accord with previous studies [43]. The current study also found that reporting a previous suicide attempt was associated with the risk of suicide, which was consistent with several examples of previous research [44]. In fact, reporting a previous suicide attempt and being separated or being widow were the variables with a major contribution to risk of suicide according to the Table 4.

The regression analysis revealed that the presence of avoidant, borderline, schizotypal and depressive are all associated with the risk of suicide. These results are broadly consistent with previous research [3,45,46,47,48]. A diagnosis of borderline PD doubled the risk of suicide when compared to patients diagnosed with other types of PD [38]. Links and his collaborators [47] found that 25.6% of participants with borderline PD attempted suicide during the course of one year of treatment. Moreover, 60% to 70% of patients with borderline PD reported a history of suicidal behavior [49]. These relationships may partly be explained by the role of impulsivity as a key background factor [3]. 

PDs are relevant factors to take into account related to the risk of suicide among SUD patients. As a practical implication of the present findings, the results indicate that the identification of comorbidity of SUD is important for improving the treatment among the bereaved drug-dependent population as well as reducing suicidal ideation because, as Schneider et al. noted [40], treating PDs is essential for suicide prevention.

The current research presented some limitations that should be considered. It is important to mention that this study was performed using a convenience sample of bereaved substance users, who attended a drug addiction treatment center. Therefore, our sample might be different from the general drug user population. Furthermore, the current research had a cross-sectional design and we relied on self-reporting measures. Thus, we must be cautious due to the small number of PD cases according with the most conservative scoring of Millon [36]. Notwithstanding these limitations, this study provided significant data related to the specificity of the sample. Clarifying the pattern of risk across mental disorders is a necessary step to identify where resources can be most effectively targeted and interventions prioritized [50]. 

## 5. Conclusions

To date, there are no studies that have investigated the association between risk of suicide and dysfunctional patterns of personality among a bereaved SUD sample. As predicted, reporting PDs are linked with the risk of suicide, with the exception of the narcissistic scale. The presence of avoidant, depressive, schizotypal and borderline personality disorders are associated with the risk of suicide. In conclusion, these findings outline the importance of performing therapeutic interventions in order to focus on PD in those bereaved SUD patients and to reduce and prevent the risk of suicide. 

## Figures and Tables

**Table 1:** Relationship between personality disorders (PDs) and risk of suicide.

Personality Disorder	Presence/Absence of Disorder	Presence of Disorder (*n*, (%))	No Risk of Suicide	Risk of Suicide	*p*
Schizoid	no disorder		76 (100)	118 (98.3)	0.523
	disorder	2 (1)	0	2 (1.7)	
Avoidant	no disorder		76 (100)	120 (100)	-
	disorder	0	-	-	
Depressive	no disorder		76 (100)	119 (99.2)	1.000
	disorder	1 (0.5)	0	1 (0.8)	
Dependent	no disorder		76 (100)	120 (100)	-
	disorder	0	-	-	
Histrionic	no disorder		73 (96.1)	120 (100)	0.057
	disorder	3 (1.5)	3 (3.9)	0	
Narcissistic	no disorder		70 (92.1)	112 (93.3)	0.781
	disorder	14 (7.1)	6 (7.9)	8 (6.7)	
Antisocial	no disorder		74 (97.4)	113 (94.2)	0.487
	disorder	9 (4.6)	2 (2.6)	7 (5.8)	
Sadistic	no disorder		76 (100)	114 (95)	0.084
	disorder	6 (3.1)	0	6 (5)	
Compulsive	no disorder		67 (88.2)	115 (95.8)	0.050
	disorder	14 (7.1)	9 (11.8)	5 (4.2)	
Negativist	no disorder		76 (100)	118 (98.3)	0.523
	disorder	2 (1)	0	2 (1.7)	
Masochistic	no disorder		76 (100)	120 (100)	-
	disorder	0	-	-	
Schizotypal	no disorder		76 (100)	119 (99.2)	1.000
	disorder	1 (0.5)	0	1 (0.8)	
Borderline	no disorder		76 (100)	118 (98.3)	0.523
	disorder	2 (1)	0	2 (1.7)	
Paranoid	no disorder		75 (98.7)	117 (97.5)	1.000
	disorder	4 (2)	1 (1.3)	3 (2.5)	

**Table 2:** Mann–Whitney *U* test of PD symptoms related to presence of risk of suicide (M (SD)).

MCMI-III Scales	No Risk	Risk of Suicide	*U*	*Z*
Schizoid	7.07 (3.68)	10.88 (4.73)	<0.001	−5.448
Avoidant	6.58 (4.47)	9.73 (5.64)	<0.001	−3.677
Depressive	5.87 (4.84)	12.21 (5.59)	<0.001	−7.051
Dependent	7.57 (4.73)	10.07 (4.91)	0.001	−3.237
Histrionic	15.03 (4.14)	12.34 (5.10)	<0.001	−3.610
Narcissistic	14.72 (4.06)	14.17 (4.65)	0.410	−0.824
Antisocial	9.93 (5.23)	12.94 (5.14)	<0.001	−3.783
Sadistic	9.46 (5.13)	13.30 (5.36)	<0.001	−4.710
Compulsive	17.04 (4.26)	14.97 (4.48)	0.002	−3.157
Negativist	8.67 (4.84)	13.36 (5.79)	<0.001	−5.487
Masochistic	3.71 (3.44)	8.12 (4.55)	<0.001	−6.486
Schizotypal	4.84 (4.67)	9.44 (5.86)	<0.001	−5.586
Borderline	5.87 (12.43)	3.94 (5.46)	<0.001	−7.768
Paranoid	8.2 (5.64)	11.30 (6.26)	0.001	−3.382

**Table 3:** Relationship between symptomatology of PD and risk of suicide through the Spearman’ correlation.

MCMI-III Scales	Spearman’s Correlation	*p*
Schizoid	0.492 *	<0.001
Avoidant	0.334 *	<0.001
Depressive	0.628 *	<0.001
Dependent	0.354 *	<0.001
Histrionic	−0.324 *	<0.001
Narcissistic	−0.072	0.314
Antisocial	0.347 *	<0.001
Sadistic	0.417 *	<0.001
Compulsive	−0.310 *	<0.001
Negativist	0.493 *	<0.001
Masochistic	0.529 *	<0.001
Schizotypal	0.523 *	<0.001
Borderline	0.703 *	<0.001
Paranoid	0.320 *	<0.001

* Correlation is significant in the level 0.01.

**Table 4:** Multiple regression analysis of dysfunctional patterns of personality associated with risk of suicide.

	Unstandardized Coefficients		Standardized Coefficients	*t*	*p*	95% Confidence Interval for B	
	B	Standard Error	Beta			Lower Bound	Upper Bound
Constant	6.289	2.100		2.995	0.003	2.144	10.433
Age	0.004	0.017	0.013	0.263	0.793	−0.029	0.037
Gender	0.239	0.366	0.029	0.653	0.515	−0.483	0.961
Education	0.441	0.338	0.060	1.304	0.194	−0.227	1.108
Marital status							
Single	0.125	0.404	0.015	0.310	0.757	−0.672	0.922
Separated	1.235	0.353	0.171	3.498	0.001	0.538	1.931
Widow	2.612	0.579	0.208	4.514	<0.001	1.470	3.754
Patient’s suicide attempt	−2.162	0.353	−0.299	−6.116	<0.001	−2.860	−1.464
Schizoid	0.077	0.055	0.108	1.421	0.157	−0.030	0.185
Avoidant	−0.118	0.054	−0.189	−2.200	0.029	−0.223	−0.012
Depressive	0.135	0.045	0.243	3.033	0.003	0.047	0.223
Dependent	−0.021	0.043	−0.030	−0.477	0.634	−0.106	0.065
Histrionic	−0.049	0.057	−0.071	−0.856	0.393	−0.162	0.064
Narcissistic	−0.031	0.048	−0.041	−0.657	0.512	−0.126	0.063
Antisocial	−0.042	0.051	−0.067	−0.823	0.412	−0.143	0.059
Sadistic	0.030	0.049	0.050	0.616	0.539	−0.066	0.127
Compulsive	−0.024	0.047	−0.032	−0.519	0.604	−0.117	0.068
Negativist	0.032	0.049	0.056	0.658	0.511	−0.065	0.130
Masochistic	0.046	0.059	0.063	0.781	0.436	−0.070	0.162
Schizotypal	0.104	0.049	0.179	2.101	0.037	0.006	0.202
Borderline	0.169	0.058	0.292	2.932	0.004	0.055	0.283
Paranoid	−0.050	0.043	−0.091	−1.170	0.244	−0.134	0.034
